# Prevalence and Characteristics of Non-tuberculous Mycobacteria (NTM) Infection in Recipients of Allogeneic Hematopoietic Stem Cell Transplantation: a Systematic Review and Meta-analysis

**DOI:** 10.1007/s10875-023-01615-3

**Published:** 2023-12-22

**Authors:** Bianca Laura Cinicola, Giorgio Ottaviano, Ilie Fadzilah Hashim, Zarina Thasneem Zainudeen, Intan Juliana Abd Hamid, Reem Elfeky

**Affiliations:** 1 Department of Maternal Infantile and Urological Sciences, Sapienza University of Rome, Rome, Italy; 2 Department of Molecular Medicine, Sapienza University of Rome, Rome, Italy; 3 Department of Pediatrics, Fondazione IRCCS San Gerardo dei Tintori, Monza, Italy; 4 Primary Immunodeficiency Diseases Group, Department of Clinical Medicine, Advanced Medical and Dental Institute, Universiti Sains Malaysia, Bertam, 13200 Kepala Batas, Pulau Pinang Malaysia; 5 Department of Immunology, Great Ormond Street Hospital for Children NHS Foundation Trust, Great Ormond Street, London, UK; 6 GOS Hospital for Children NHS Foundation Trust, University College London GOS Institute of Child Health, and NIHR GOSH BRC, London, UK

**Keywords:** Non-tuberculous mycobacteria, NTM, Hematopoietic stem cell transplant, HSCT, Immunodeficiency, Infection

## Abstract

**Purpose:**

Non-tuberculous mycobacteria (NTM) infections in hematopoietic stem cell transplantation (HSCT) recipients represent a diagnostic and therapeutic challenge. Here, we aimed to review and analyze current literature on incidence, clinical presentation, and outcome of NTM infection after allogeneic HSCT.

**Methods:**

We performed a systematic review and meta-analysis of available literature regarding NTM infection in children and adults receiving allogeneic HSCT.

**Results:**

We identified 56 articles eligible for the analysis. Among 15 studies, describing 15,798 allogeneic HSCT, we estimated a prevalence of 1.26% (95% CI 0.72, 1.93) of NTM after transplant. Analysis of 175 patients with NTM infection showed a median time of diagnosis of 318 days after HSCT, an increased prevalence in adults (82.9%), and a most frequent pulmonary involvement (44%). Comparison between children and adults revealed an earlier post-transplant disease onset (median 130 days vs 287 days) and most frequent non-pulmonary presentation in children. A vast heterogeneity of therapeutic approach reflected the lack of universal recommendations regarding drug combination and duration of therapy. Overall, NTM-related mortality accounted for 33% in this systematic review.

**Conclusion:**

Although rare, NTM infections can complicate post-transplant course with a high mortality rate in children and adults. The lack of prospective studies and guidelines prevents identification of risk factors and therapeutic recommendations.

**Supplementary Information:**

The online version contains supplementary material available at 10.1007/s10875-023-01615-3.

## Introduction

Infections are among the most frequent complications occurring in patients receiving hematopoietic stem cell transplant (HSCT) [1]. As diagnostic techniques advanced, specific cultures are now able to identify a wide spectrum of pathogens causing infections post-HSCT, including non-tuberculous mycobacteria (NTM) [2]. Mycobacteria are responsible of several infectious manifestations in transplant recipients. However, while tuberculous mycobacteria infections/reactivation or Bacillus Calmette-Guerin (BCG) vaccine-associated complications have been widely described [3], there is limited data on the prevalence, clinical presentation, outcome, and risk factors associated with NTM infection post-HSCT. NTM are ubiquitous organisms that grow and persist in natural environments including water, household plumbing systems, soil, dust, foods, and a variety of animals [4]. More than 170 NTM species have been identified, most of them being commensal organisms colonizing skin, respiratory, and gastrointestinal tract in humans [5]. Although NTM are rarely pathogenic in immunocompetent subjects, approximately 20 NTM species have been reported as causative pathogens [6] especially in immunocompromised hosts [7].

Human disease due to NTM has been generally classified also into two distinct clinical manifestations: pulmonary disease and extra-pulmonary (including lymph node, cutaneous, soft tissue infections, joints, and gut infection) [8, 9]. Disseminated disease affecting more than one organ was seen, mainly among immunocompromised recipients [10, 11].

NTM pathogenicity depends on different factors namely site of infection, species-specific virulence, host factors, and interactions between the latter two [12]. The main prominent factors that increase the risk of NTM infection include pre-existing structural lung disease and genetic or secondary impairment of cell-mediated immunity [13]. In fact, a functional IL-12/23-IFN-γ integrity and a good interaction and cooperation between macrophages and T and natural killer (NK) lymphocytes are essential to control mycobacterial infections [11, 14, 15]. Recipients of HSCT have an impaired immune system with a noted delay in the immune reconstitution of T cells and cell-mediated immunity, in comparison to innate and B cell immunity, regardless of the source of stem cells [16]. Such a cohort is thus susceptible to NTM infection and is at risk of disseminated disease [17, 18]. Moreover, these patients might require immune suppressive therapy to control autoimmunity or graft versus host disease, increasing their risk for disseminated NTM infection [19]. It remains difficult to discern HSCT recipients who are at highest risk for NTM disease. Allograft T cell depletion and the allogeneic HSCT conditioning regimen (e.g., total body irradiation (TBI)) are potential risk factors for NTM infections [20].

Diagnosis of NTM in HSCT recipients is considered challenging with difficulty in distinguishing colonization from active infection and between infection and other infectious or non-infectious complications related to HSCT, leading to a similar clinical picture (e.g., lung GvHD) [17, 20, 21]. A high index of suspicion and improvement in diagnostic techniques are thus mandated for proper diagnosis and initiation of treatment for better outcome post-HSCT.

Moreover, the decision to start a treatment for NTM-associated disease is not easy among this cohort of patients, since multidrug regimens are required, with numerous toxicities and the potential metabolic interaction with other treatments [22]. Also, the optimal treatment and duration for NTM infections in HSCT recipients remain undefined [23].

This is a systematic review and meta-analysis research focusing on the prevalence and characteristics of NTM infection in recipients of allogeneic hematopoietic stem cell transplantation, with the aim to clarify the most encountered species, sites of disease, NTM-related mortality and differences between adults and pediatrics.

## Methods

This is a systematic review and meta-analysis research focusing on NTM infection in recipients of allogeneic HSCT. A literature search was performed for all studies associated with NTM infections post-HSCT. Two reviewers independently extracted the data and assessed the quality of the studies. Meta-analyses of pre-specified outcomes were performed when deemed appropriate. The PICO statement was as follows: patient/population (P) = patients underwent allogeneic hematopoietic stem cell transplantation; intervention or exposure (I) = diagnosed with NTM infection after the transplantation; comparison or control (C) = children and adult cohort; outcomes (O) = prevalence, NTM infections, and risk factors.

### Search Strategy

We performed a comprehensive systematic search of articles published in peer reviewed journals using PubMed/MEDLINE (from 1983 until 2020). A structured search was conducted with controlled vocabulary and relevant key terms to enhance sensitivity. Reference lists of included papers were hand searched for additional relevant studies. The search was restricted to articles in English language.

### Study Selection

Two investigators performed an initial screening of identified titles and abstracts. Those deemed to be clearly irrelevant were removed on the initial screen. They also performed a second screen to identify potentially relevant studies. If no abstract was available, the full text was obtained unless the article could be confidently excluded by title alone. If there was any doubt as to whether or not a study could be excluded, a full-text screen was performed to reduce the likelihood of incorrect exclusion of a relevant study. Three reviewers obtained and independently screened full-text versions of potentially eligible studies to determine their eligibility based on the selection criteria. Any disagreements during the screening process were resolved through discussion among the authors in accordance with the selection criteria. We included cross-sectional, retrospective cohort studies that reported NTM infection in allogeneic HSCT recipients. To be included in the analysis, records must have described patients receiving allogeneic HSCT, without known pre-existing NTM infection. Studies describing patients with Mendelian susceptibility to mycobacterial disease (MSMD) or with NTM colonization (e.g., NTM infection detected in asymptomatic patients as infection screening) were also excluded.

### Data Extraction and Synthesis

Three investigators independently extracted the data from each eligible study. Assessed variables related to the organization and outcome of the studies included study design, the year of reporting, countries, age, gender, number of study participants, NTM species, the timing to NTM infections in post-allogeneic transplant, and reporting of relevant outcomes. The primary objective was to estimate cumulative prevalence of NTM after allogeneic HSCT. The secondary objectives were to determine the characteristics of NTM infection post-HSCT and to assess the risk factors associated with NTM post HSCT. To calculate the prevalence of NTM among HSCT recipients, only studies reporting the total number of patients who have received allogeneic HSCT were thus included. Disseminated NTM was defined as including either blood stream infection or more than one organ affected with or without blood stream infection. The data analysis was performed by 3 investigators.

### Patient Involvement

There was no patient involvement in this study.

### Statistical Analysis

Descriptive methods were used to present the data by year of study, country, total transplant, total NTM patients, and prevalence based on reported study. Meta-analyses were performed using STATA SE16 software. Data from case studies were not pooled for prevalence study because of concerns about the validity of the results. The reporting of this systematic review is in accordance with PRISMA guidelines.

## Results

### Characteristics of Included Studies

**Fig. 1:**
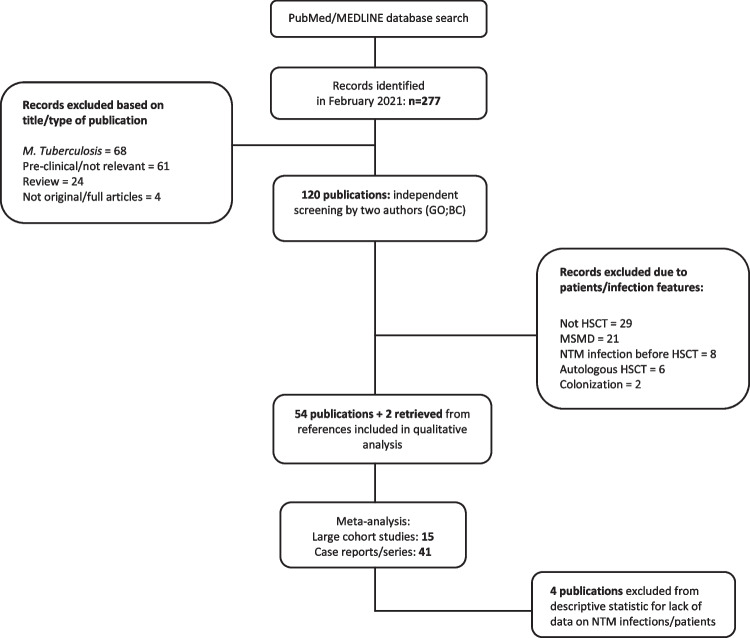
PRISM flow chart for the systematic review

A total of 277 records were identified in February 2021.We excluded studies that showed only non-clinical data (in vitro studies or those performed on animal models) (*n* = 61), review articles (*n* = 24), articles on mycobacteria tuberculosis infection rather than NTM (*n* = 68) besides studies with non-original or incomplete data (*n* = 4). One hundred twenty publications were thus screened independently by 2 authors. A further 66 studies were excluded; NTM infections in autologous rather than allo-HSCT setting (*n* = 6), NTM infection but in non-HSCT setting (*n* = 29), reported NTM colonization rather than infection (*n* = 2), NTM infection acquired pre-HSCT (*n* = 8), and reports of NTM infection in patients with Mendelian Susceptibility to Mycobacteria Disease (MSMD) (*n* = 21). Overall, 54 publications were considered eligible for the analysis, and two further records were retrieved from the references of included articles (Fig. 1). The final analysis was conducted on 56 publications, divided in two categories: (i) large cohort studies (≥ 90 HSCT transplant recipients), included in the prevalence analysis, and (ii) case report/series.

### The Prevalence of NTM Infection Among Allogeneic HSCT Recipients

**Fig. 2:**
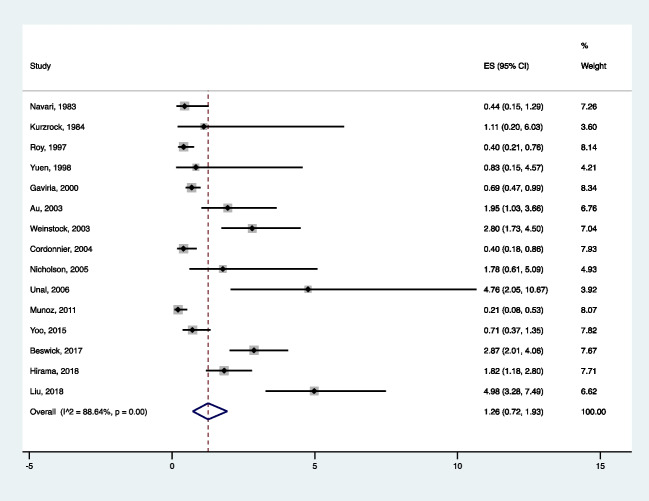
Forest plot of the meta-analysis on prevalence of post-HSCT NTM infections

**Fig. 3:**
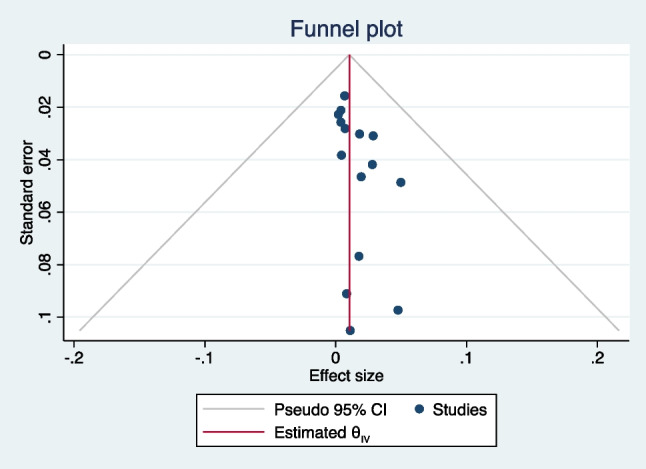
Funnel plot of the meta-analysis on prevalence of post-HSCT NTM infections

For the meta-analysis calculation for the prevalence of NTM post-HSCT, 15 studies fulfilled the inclusion criteria and were included in the analysis [19–21, 24–35] (Fig. 1, Supplementary tables 1 and 2). A total of 15,798 patients who underwent allogeneic HSCT were evaluated. The combined prevalence of NTM post-HSCT was 1.26% (95% CI 0.72, 1.93) (Fig. 2), estimated via random effect model. The heterogeneity of the included studies was *I*^2^ = 88.64*%, p* < 0.001. The Egger’s weighted regression test analysis suggested no small study effect noted in the meta-analysis (*t* = 0.72, *p* = 0.47) and there was no apparent bias in the studies included in the meta-analysis (Fig. 3).

### The Characteristics of NTM Infections Post-HSCT

A total of 175 cases of NTM infection post-allogeneic HSCT in 52 publications [both types of reporting (case reports and cohort studies)] were included in the analysis [20, 21, 24–27, 29, 31–33, 35–76]. Four publications (total studies 56) were ultimately excluded given lack of data on patients and NTM infection characteristics [19, 28, 30, 34]. All the papers were published between 1983 until 2020. The median age of reported cases was 35 years old (range 1–69 years old). Thirty cases (17.1%) were patients aged less than 18 years old, and 145 cases (82.9%) involved patients aged equal or more than 18 years old. Seventy-nine patients (45.1%) were female, and in 5%, gender was not reported. Cases were reported mainly from North America (110 cases, 62.9%), followed by from Asia (48 cases, 27.4%), Europe (10 cases, 5.7%), and Australia (7 cases, 4%).

With regards to the NTM species, the most frequently grown species were *Mycobacterium Avium Complex* (MAC) followed by *M. haemophilum*, *M. chelonae*, *M. fortuitum*, and *M. abscesses*. The timing of NTM infections in post-allogeneic transplant recipients varied according to the type of species as shown in Table 1. In most species, NTM was diagnosed at a median of 318 days post-HSCT.

**Table 1:** Time interval from HSCT to the diagnosis of NTM based on species

Species	Timing to NTM infections post allogeneic transplantation (days) Median (min, max)	Number of patients
*MAC*	364 (14, 3650)	26
*M. haemophilum*	123 (90, 1460)	19
*M. chelonae*	422 (7, 1642)	18
*M. abscessus*	381 (10, 3285)	14
*M. fortuitum*	301 (50, 1140)	14
*M. kansasii*	120 (35, 1270)	5
*M. immunogenum*	140 (130, 180)	4
*M. xenopi*	306 (133, 649)	4
*M. genavense*	264 (240, 253)	2
*M. massiliense*	946 (3, 1890)	2
*M. gordonae*	120	1
*M. marinum*	174	1
*M. mucogenicum*	180	1
*M. neoaurum*	2	1
*M. szulgai*	84	1
*M. scrofulaceum*	2040	1

*M. Mycobacterium*, *MAC Mycobacterium avium* complex

Most of the reported patients (77/175; 44%) had pulmonary NTM (with no other organ involvement) with MAC being the most frequently reported organism (23%). Forty-three patients (24.5%) had cutaneous NTM (with no other organ involvement); most predominant organism retrieved was *M. haemophilum* grown in 29 patients (67%). Disseminated NTM was seen in 38 patients (21.7%) with no noted predominant species (Table 2). Few cases had other sites of NTM infection including gastrointestinal in 6 patients, catheter related in 4 patients, musculoskeletal in 2 patients, and lymph node infiltration in 1 patient.

**Table 2:** NTM site of infection and related species

Infection sites	Number of patients affected for each species/number of total cases
Pulmonary [20, 21, 25, 29, 32, 33, 35, 36, 38, 40, 41, 44, 48, 49, 59, 62, 66–68, 76]	77/175 (44%) MAC, 18 *M. abscessus*, 10 *M. chelonae*, 8 *M. haemophilum*, 8 *M. fortuitum*, 6 *M. xenopi*, 4 *M. kansasii*, 2 *M. fortuitum* and *M. chelonae, 1* *M. massiliense*, 1 *M. scrofulaceum*, 1 MAC and *M. gordonae*, 1 Unclassified, 17
Cutaneous [20, 24, 26, 31, 32, 37, 41–43, 45, 46, 50–52, 55, 58, 61, 63, 71, 76]	43/175 (24.6%) *M. haemophilum*, 29 *M. chelonae*, 4 *M. fortuitum*, 3 *M. kansasii*, 2 *M. abscessus*, 1 *M. marinum*, 1 *M. massiliense*, 1 *M. szulgai*, 1 MAC, 1
Disseminated [20, 26, 29, 31, 32, 36, 38, 40, 42, 47, 53, 57, 59, 64, 69, 72–74, 76]	38/175 (21.7%) *M. mucogenicum*, 8 *M. fortuitum*, 5 *M. haemophilum*, 5 *MAC*, 4 *M. chelonae*, 4 *M. immunogenum*, 4 *M. abscessus*, 3 *M. fortuitum* and *M. haemophilum, 1* M. genavense, 1 *M. gordonae*, 1 *M. massiliense*, 1 *MAC and M. haemophilum*, 1
Others [29, 31, 35, 39, 54, 56, 59, 60, 65, 70, 74, 76]	15/175 (8.6%) MAC, 4 *M. chelonae*, 4 *M. haemophilum*, 2 *M. abscessus*, 1 *M. genavense*, 1 *M. neoaurum*, 1 Unclassified, 2
Musculoskeletals [35, 75]	2/175 (1.1%) *M. kansasii*, 2

*M*. *mycobacterium*, *MAC Mycobacterium avium* complex

NTM-directed therapy was initiated for 138 patients (78%), including at least two drug combinations. Data on specific treatment was not available for 18 reported cases. Moreover, 19 patients did not receive any NTM-directed therapy (10%): 4 of them passed away (1 died before initiating therapy and 3 had the diagnosis identified post-mortem) and 1 patient had resolution of infection after line removal. In the remaining 14 cases, the cause for not initiating NTM-directed therapy was not clear: 8 of them were alive at time of reporting while the other 6 patients died from NTM infection (data not shown). Median duration of therapy among 92 cases (where length of therapy was mentioned) was 9.2 months.

A total of 60 deaths (40%) were reported at the time of publications with NTM infection being the cause of death in 20 patients (33% of the total NTM cohorts).

### Differences in NTM Infection Between Pediatric and Adult Cohort

For reported NTM cases affecting children, the median age at presentation was 9 years old (range 1.2–17 years old); the majority of patients were boys (18 cases, 60%). An equal number of cases received HSCT for malignant or non-malignant disease. Most patients received an HLA-matched graft (11 cases, 36.7%). Unfortunately, the type of graft source, conditioning regimens, and the presence of acute GVHD were not documented in most of the publications. The majority of this age group had non-disseminated (66.7%) and non-pulmonary (66.7%) NTM infection. Slowly growing NTM species were isolated in 53.3%. The most frequently isolated NTM species reported was MAC (8 cases, 26.7%) followed by *M. chelonae* (4 cases, 13.3%) and *M. kansasii* (4 cases, 13.3%). Five patients were reported to be dead at the time of publication of the articles (16.7%).

For reported NTM cases affecting adults, the median age at presentation was 42 years old (range 18–69). There were no gender differences noted, and the majority had a malignant disease as indication for HSCT (89%). Unfortunately, as seen among paediatric publications, the type of graft source, conditioning regimens, and the presence of acute GvHD were not documented in the majority of publications. The majority of adults—similarly to children—had a non-disseminated NTM infection (79.3%). However, in contrast with the paediatric cohort, there was no difference between pulmonary versus non-pulmonary NTM infection proportions among adults. *M. haemophilum* was the most reported species in this cohort (44 cases, 30.3%) followed by MAC (19 cases, 13.1%), *M. chelonae* (16 cases, 11%), *M. abscessus* (13 cases, 8.9%), and *M. fortuitum* (11 cases, 7.6%).

The time to NTM infection post-HSCT was noted to be longer among adults in comparison to children (median 287 days vs 130 days). The clinical characteristics of the NTM infections post-HSCT are listed in Table 3 and in details in Supplementary Table 2.

**Table 3:** Clinical characteristics of NTM infections among reported allogeneic HSCT recipients from 1983 to 2020

Characteristics	Children *n* = 30	Adults *n* = 145	Total *n* = 175
Age in years			
Mean (SD)	8.7 (4.5)	40.9 (13.3)	
Median (min, max)	9 (1.2, 17)	42 (18, 69)	
Gender (*n*, %)			
Male	18 (60%)	69 (47.6%)	87 (50%)
Female	11 (36.7%)	68 (46.9%)	79 (45%)
Not available	1 (3.3%)	8 (5.5%)	9 (5%)
Diagnosis upon HSCT (*n*, %)			
Malignant	15 (50%)	129 (89%)	144 (82%)
Non-malignant	15 (50%)	8 (5.5%)	23 (13%)
Not available	-	8 (5.5%)	8 (5%)
HLA matching (*n*, %)			
Matched	11 (36.7%)	65 (44.9%)	76 (43%)
Mismatched	3 (10%)	6 (4.1%)	9 (5%)
Not available	16 (53.3%)	74 (51%)	90 (52%)
Graft source (*n*, %)			
BM	2 (6.7%)	6 (4.1%)	8 (5%)
PBSC	1 (3.3%)	5 (3.5%)	6 (3%)
UCB	6 (20%)	1 (0.7%)	7 (4%)
Not available	21 (70%)	133 (91.7%)	154 (88%)
Conditioning (*n*, %)			
Myeloablative	5 (16.7%)	64 (44.1%)	69 (40%)
Non-myeloablative	3 (10%)	3 (2.1%)	6 (3%)
Not available	22 (73.3%)	78 (53.8%)	100 (57)
Severe acute GVHD (*n*, %)			
Yes	11 (36.7%)	60 (41.4%)	71 (41%)
No	5 (16.7%)	24 (16.6%)	29 (17%)
Not available	14 (46.6%)	61 (42%)	75 (42%)
Location of NTM isolated (*n*, %)			
Disseminated	10 (33.3%)	30 (20.7%)	40 (23%)
Non-disseminated	20 (66.7%)	115 (79.3%)	135 (77%)
Pulmonary	10 (33.3%)	72 (49.7%)	82 (47%)
Non-pulmonary	20 (66.7%)	73 (50.3%)	93 (53%)
Types of NTM (*n*, %)			
Slowly growing NTM	16 (53.3%)	74 (51%)	90 (52%)
Rapidly growing NTM	14 (46.7%)	52 (35.9%)	66 (38%)
Unclassified	-	19 (13.1%)	19 (10%)
NTM species (n, %)			
*M. abscessus*	2 (6.7%)	13 (8.9%)	15 (9%)
*M. chelonae*	4 (13.3%)	16 (11%)	20 (11%)
*M. fortuitum*	3 (10%)	11 (7.6%)	14 (8%)
*M. genavense*	1 (3.3%)	1 (0.7%)	2 (1%)
*M. gordonae*	-	1 (0.7%)	1 (0.5%)
*M. haemophilum*	-	44 (30.3%)	44 (25%)
*M. immunogenum*	3 (10%)	1 (0.7%)	4 (2%)
*M. kansasii*	4 (13.3%)	2 (1.4%)	6 (3%)
*M. marinum*	-	1 (0.7%)	1 (0.5%)
*M. massiliense*	-	3 (2.1%)	3 (1.5%)
*M. mucogenicum*	2 (6.7%)	6 (4.1%)	6 (3%)
*M. neoaurum*	1 (3.3%)	-	1 (0.5%)
*M. szulgai*	1 (3.3%)	-	1 (0.5%)
*MAC*	8 (26.7%)	19 (13.1%)	27 (15%)
*M. xenopi*	-	4 (2.8%)	4 (2%)
*M. scrofulaceum*	-	1 (0.7%)	1 (0.5%)
*MAC* and *M. gordonae*	1 (3.3%)	-	1 (0.5%)
*M. fortuitum* and *M. chelonae*	-	1 (0.7%)	1 (0.5%)
*M. fortuitum* and *M. haemophilum*	-	1 (0.7%)	1 (0.5%)
*MAC* and *M. haemophilum*	-	1 (0.7%)	1 (0.5%)
*Unclassified*	-	19 (13.1%)	19 (10%)
Timing to NTM infections post allogeneic transplantation (days) Median (min, max)	130 (2,1642)	287 (3,3650)	
Outcome summary			
Alive	20 (66.7%)	68 (46.9%)	88 (51%)
Dead	5 (16.7%)	55 (37.9%)	60 (34%)
Missing data	5 (16.7%)	22 (15.2%)	27 (15%)

*HSCT* hematopoietic stem cell transplant, *HLA* human leukocyte antigen, *BM* bone marrow, *PBSC* peripheral blood stem cell, *UCB* umbilical cord blood, *GVHD* graft versus host disease, *NTM* non-tuberculous mycobacteria, *M*. *Mycobacterium*, *MAC Mycobacterium avium* complex

## Discussion

To our knowledge, this is the first meta-analysis study investigating prevalence and characteristics of NTM infection post-allogeneic HSCT for both paediatric and adult cohorts. We reported a combined prevalence rate of NTM disease of 1.6%. This is significantly higher than what has been reported so far in both immunocompetent hosts and in patients with human immunodeficiency virus (HIV). Among immunocompetent hosts, NTM disease prevalence was noted to be 0.2–3.1 (2.9 in UK [77]) in Europe, in 8.6–9.08 in USA and Canada, and in Brazil is 0.25/100,000 individuals [78].

In pre-antiretroviral therapy (ART) era, it was reported that up to 43% of AIDS patients had disseminated NTM infections, caused in the 86% of cases by MAC [79]. Following the introduction of both ART and primary prophylaxis, the overall incidence of disseminated MAC infection in HIV-infected patients has decreased to about 2.5 cases per 1000 person-years [80], which is still far less than what is reported in our study.

Interestingly, in our meta-analysis, we noted more cases reported from North America (almost 60%) followed by Asia (27%) in comparison to few cases (< 10%) reported from Australia and Europe and none from Africa or South America. While this might reflect environmental, climate prevalence of NTM, there might be other factors. In resource-limited settings, diagnosis of NTM could be missed because of scarce access to adequate laboratory and differentiation between TB and NTM might be a challenge [81]. Patients are empirically treated as drug sensitive or resistant TB [82] rather than for NTM infection.

In the allogeneic HSCT setting, multiple factors could be responsible for the development of NTM: including impaired cell-mediated immunity with the use of serotherapy like ATG, TBI-based conditioning (associated with lymphotoxicity), during early phases before immune recovery in the context of GvHD, and the use of immune suppressive medications to prevent or treat GvHD[17, 20, 83]. Unfortunately, the graft source, conditioning regimens, in vivo lymphodepletion, and GvHD were not reported in most of the included studies; thus, we are not able to evaluate the role of these factors on the development of NTM among the reported cases.

NTM infection occurs mainly by inhalation, ingestion, or direct inoculation, leading to different sites of colonization [83]. Lymphadenitis is the most common manifestation of NTM disease in healthy childhood [6, 84] while pulmonary NTM is mainly seen in patients with underlying lung pathology such as cystic fibrosis, bronchopulmonary dysplasia, or primary ciliary dyskinesia [6, 8].

In the immunosuppressed population, extrapulmonary NTM disease is more common [11]. The infection can occur at any site and then become disseminated because the organisms that escape intracellular killing are able to multiply within macrophages and disseminate via lymphatics and the bloodstream (84).

In our study, the majority of cases were adults rather than pediatrics, with only one-fifth of the reported cases being 18 years old or less. Interestingly, rates of disseminated NTM were 33% and 20.7% among pediatrics and adults, respectively, with no predominant causative pathogen. We noted a difference in the timing of NTM infection post-HSCT, between the pediatric and adult cohort where NTM occurred earlier among children rather than adults (4 months vs 9 months post-HSCT). There is no clear explanation for this observation. The late occurrence of NTM among adults might be in part related to impaired recovery of cell-mediated immunity in adults in the context of thymus involution by age and need for thymus gland to support T cell development, maturation, and education [85, 86]. Also, as in adults the majority of patients received HSCT for malignant conditions, impact of pre-HSCT chemotherapy might have contributed to a slower immune recovery. Again, the lack of extensive details on conditioning, donor, stem cell source, and GvHD prophylaxis, precludes any further comparison between the two cohorts.

There are multiple guidelines set for adults to support a diagnosis of NTM including the American and British Thoracic Societies guidelines [87–89]. However, none of these are validated in children. Across the selected studies and case reports, we noticed that clinicians relied on clinical and pathogen recognition rather than using any of the published guidelines. Finally, most likely because we excluded patients with MSMD and pre-transplant NTM infections, no cases of immune reconstitution inflammatory syndrome (IRIS), that could guide diagnostic and therapeutic considerations, were reported in the analyzed studies.

In general, empiric treatment is usually begun before results on susceptibility testing are available [90]. Treatment of NTM disease requires the use of two or more antimicrobial agents for prolonged periods, to achieve microbiologic cure or radiologic improvement while avoiding the emergence of resistant strains [23]. The choice of antimicrobial therapy for transplant recipients is similar to that for patients without systemic immune suppression. However, in addition to well-documented toxicities and interactions of drugs used to treat NTM infection, interactions between medications for NTM infection and immunosuppressive agents (and other medications) are frequent and need to be considered especially in a transplant setting [18]. Moreover, a careful evaluation of the balance between risks and benefits is warranted for each patient, taking into account the clinical course of the disease, the pathogenicity of the isolated NTM species, and the underlying pathologies of the patient [22]. Several schedules of treatment are employed to contrast NTM infections in immunocompromised patients based on combination of antitubercular drugs, macrolides, aminoglycosides, and quinolones. International guidelines for therapy in adults with NTM lung infections have been published, while definite management in immunocompromised children has not yet been formulated and the optimal regimen is yet to be determined [91].

There is no clear recommendation for duration of treatment for NTM infection in the immunocompromised host, including HSCT recipients [23]. Besides antimicrobial NTM-targeted therapy, other interventions including foreign body removal in catheter-related infections and surgical resection or debridement of infected collections or devitalized tissue should be achieved early to allow improved penetration of drugs, to decrease the burden of disease, and to minimize the risk of developing resistance [29, 39, 42, 45, 53, 65, 69, 73, 74].

There are scarce data regarding outcome of NTM infections and related mortality among HSCT recipients. In our study, data on the patient’s status at time of reporting was missing for 27 cases. Among the remaining cases; 88 patients (88/145; 59%) were alive at time of reporting. Mortality rate of 40%, attributed to NTM in 33% of cases, is similar to what has been reported among patients with NTM infection in the setting of HIV in the pre-ART era. [92].

There are a number of limitations for this study including heterogeneity of the data and possible publication bias where some species tend to be reported more. Moreover, all analyzed studies only reported retrospective data on NTM infections, and differences in diagnostic procedures might have contributed to different incidences reported. Finally, our analysis was restricted to the English language literature, and therefore, we might have not included publications from Africa or South America.

## Conclusions

NTM infection is not rare among HSCT recipients and is associated with NTM-related mortality rates of 33%. Active surveillance and early initiation of therapy are mandated to improve outcome. So far, there are no guidelines on diagnosis and management of NTM infection particularly in children. Further work is needed to set guidance to support early diagnosis and active management for best outcome.

### Supplementary Information

ESM 1 (DOCX 121 kb)

## Data Availability

The raw data supporting the conclusions of the article will be made available by the authors, without undue reservation.
