# Supplementary material for: Prevalence and Characteristics of Non-tuberculous Mycobacteria (NTM) Infection in Recipients of Allogeneic Hematopoietic Stem Cell Transplantation: a Systematic Review and Meta-analysis
Source: J Clin Immunol. 2023 Dec 22;44(1):23. doi: 10.1007/s10875-023-01615-3 (PMC10739425; doi:10.1007/s10875-023-01615-3)
Supplement: Supplementary file 1 — (DOCX 121 kb) [file 10875_2023_1615_MOESM1_ESM.docx]

**Supplementary table 1: Characteristics of cohort studies included to evaluate NTM prevalence post- allogenic HSCT**

| Studies | Year of study | Country | Total transplant | Total NTM patients | Prevalence |
| --- | --- | --- | --- | --- | --- |
| Navari, 1983[24] | 1977-1982 | US | 682 | 3 | 0.1 |
| Kurzrock, 1984[25] | 1980-1984 | US | 90 | 1 | 1.1 |
| Roy, 1997[26] | 1974-1994 | US | 2241 | 9 | 0.3 |
| Yuen, 1998[27] | 1991-1993 | China | 120 | 1 | 0.8 |
| Gaviria, 2000[28] | 1977-1997 | US | 4083 | 28 | 0.7 |
| Au, 2003[29] | 1995-2002 | China | 462 | 9 | 1.9 |
| Weinstock, 2003[20] | 1993-2001 | US | 571 | 16 | 2.8 |
| Cordonnier, 2004[30] | 1994-1998 | Europe | 1513 | 6 | 0.3 |
| Nicholson, 2005[31] | 2000-2004 | US | 169 | 3 | 1.8 |
| Unal, 2006[32] | 2000-2004 | US | 105 | 5 | 4.8 |
| Munoz, 2011[33] | 1984-2008 | Spain | 1930 | 4 | 0.2 |
| Yoo, 2016[34] | 1996-2013 | South Korea | 1266 | 9 | 0.7 |
| Beswick, 2017[19] | 2001-2013 | Canada | 1047 | 30 | 2.9 |
| Hirama, 2018[21] | 2000-2013 | Canada | 1097 | 20 | 1.8 |
| Liu, 2018[35] | 2003-2014 | Taiwan | 422 | 21 | 5.0 |

**Supplementary table 2:** **Detailed description of patients with NTM infection post-allogenic HSCT**

| Paper ID/Ref | Country | Age | Sex | Background disease | HLA Matching | Source | Conditioning | Severe AGvHD/CGvHD | NTM localization | Disseminated | Pulmonary | Timing (Days) | Species | Outcome |
| --- | --- | --- | --- | --- | --- | --- | --- | --- | --- | --- | --- | --- | --- | --- |
| [36] | US | 9 | M | SCID | 10/10 | BM | BU/CY/ATG | NA | Pulmonary | N | Y | 365 | MAC | Alive  Resolved |
| [36] | US | 4 | M | SCID | HAPLO | BM | BU/CY | Y | Blood | Y | N | 1270 | MAC | Died  Other Cause |
| [37] | US | 18 | F | ALL | NA |  | NA | NA | Skin | N | N | NA | M. chelonae | NA |
| [38] | US | 27 | M | SAA | NA | NA | NA | NA | Pulmonary | N | Y | 210 | M. haemophilum | Died  Other Cause |
| [38] | US | 30 | F | AML | NA | NA | NA | NA | Blood/Skin | Y | N | 100 | M. haemophilum | Alive  Resolved |
| [39] | Australia | 17 | M | ALL | NA | NA | TBI/CY | N | Cathether | N | N | 2 | M. neoaurum | Alive  Resolved |
| [40] | US | 27 | M | NA | NA | NA | NA | NA | Pulmonary | N | Y | NA | M. haemophilum | Died  NTM Cause |
| [40] | US | 31 | F | NA | NA | NA | NA | NA | Blood/Skin | Y | N | NA | M. haemophilum | Alive  Resolved |
| [41] | US | 27 | M | AA | 10/10 | NA | TBI/CY | NA | Pulmonary | N | Y | 180 | M. haemophilum | Died  NTM Cause |
| [41] | US | 30 | F | APL | 10/10 | NA | TBI/VP16 | NA | Skin | N | N | 120 | M. haemophilum | Alive  Resolved |
| [41] | US | 29 | F | AML | 10/10 | NA | TBI/TT | NA | Skin | N | N | 120 | M. haemophilum | Alive  Resolved |
| [41] | US | 28 | F | APL | 10/10 | NA | TBI/TT | NA | Skin | N | N | 120 | M. haemophilum | Alive  Resolved |
| [41] | US | 42 | M | MDS | 10/10 | NA | TBI/TT | Y | Pulmonary | N | Y | 150 | M. haemophilum | Died  NTM Cause |
| [42] | Australia | 28 | F | AML | NA | NA | BU/CY | Y | Blood/Skin | Y | N | 90 | M. chelonae | Died_  NTM Cause |
| [42] | Australia | 26 | F | CML | 10/10 | NA | TBI/CY/ALEMT | N | Skin | N | N | 90 | M. haemophilum | Alive  Resolved |
| [43] | US | 30 | F | APL | NA | NA | NA | NA | Skin | N | N | NA | M. haemophilum | NA |
| [43] | US | 58 | M | NHL | NA | NA | NA | NA | Skin | N | N | NA | M. haemophilum | NA |
| [43] | US | 51 | F | Myeloma | NA | NA | NA | NA | Skin | N | N | NA | M. haemophilum | NA |
| [43] | US | 24 | M | AML | NA | NA | NA | NA | Skin | N | N | NA | M. haemophilum | NA |
| [43] | US | 49 | F | AML | NA | NA | NA | NA | Skin | N | N | NA | M. haemophilum | NA |
| [43] | US | 33 | F | NHL | NA | NA | NA | NA | Skin | N | N | NA | M. haemophilum | NA |
| [43] | US | 43 | M | NHL | NA | NA | NA | NA | Skin | N | N | NA | M. haemophilum | NA |
| [43] | US | 49 | F | MDS | NA | NA | NA | NA | Skin | N | N | NA | M. haemophilum | NA |
| [43] | US | 46 | M | CML | NA | NA | NA | NA | Skin | N | N | NA | M. haemophilum | NA |
| [43] | US | 28 | F | APL | NA | NA | NA | NA | Skin | N | N | NA | M. haemophilum | NA |
| [44] | India | 33 | M | CML | 10/10 | NA | BU/CY | Y | Pulmonary | N | Y | 180 | M. fortuitum and M. chelonae | Alive  Resolved |
| [45] | Japan | 30 | F | ALL | 10/10 | BM | BU/CY/TBI | Y | Skin | N | N | 900 | M. fortuitum | Alive  Resolved |
| [46] | Sweeden | 4 | M | ALL | NA | NA | NA | N | Skin | N | N | 84 | M. szulgai | Alive  Resolved |
| [47] | US | NA | NA | NA | NA | NA | NA | NA | Blood | Y | N | NA | M. mucogenicum | NA |
| [47] | US | NA | NA | NA | NA | NA | NA | NA | Blood | Y | N | NA | M. mucogenicum | NA |
| [47] | US | NA | NA | NA | NA | NA | NA | NA | Blood | Y | N | NA | M. mucogenicum | NA |
| [47] | US | NA | NA | NA | NA | NA | NA | NA | Blood | Y | N | NA | M. mucogenicum | NA |
| [47] | US | NA | NA | NA | NA | NA | NA | NA | Blood | Y | N | NA | M. mucogenicum | NA |
| [47] | US | NA | NA | NA | NA | NA | NA | NA | Blood | Y | N | NA | M. mucogenicum | NA |
| [48] | US | 29 | F | CML | NA | NA | NA | Y | Pulmonary | N | Y | 150 | M. abscessus | NA |
| [49] | US | 31 | F | ALL | 5/6 | PBSC | TBI/CY | Y | Pulmonary | N | Y | 870 | M. chelonae | Alive  Not resolved |
| [50] | Australia | 56 | M | MDS | 10/10 | PBSC | TBI/CY | Y | GI | N | N | 240 | M. genavense | Alive  Resolved |
| [51] | Spain | 25 | F | PNH | NA | NA | NA | NA | Skin | N | N | NA | M. abscessus | NA |
| [52] | Japan | 22 | M | ALL | NA | NA | NA | Y | Skin | N | N | 1825 | MAC | Alive  Resolved |
| [53] | Israel | 10 | M | Thal | NA | NA | NA | NA | Pulmonary/Blood | Y | Y | NA | M. mucogenicum | Alive  Resolved |
| [54] | Japan | 33 | M | MDS | 10/10 | BM | TBI/ARA-c | Y | GI | N | N | 80 | Unclassified | Died  Other Cause |
| [55] | US | 63 | M | AML | 10/10 | PBSC | BU/FLU/MEL | NA | Skin | N | N | 174 | M. marinum | NA |
| [56] | US | 5 | F | SCID | 4/6 | UCB | NA | NA | GI/Liver | Y | N | 256 | MAC | Died  NTM Cause |
| [57] | France | 54 | M | AML | 4/6 | UCB | BU/FLU/TT | Y | Pulmonary/Skin | Y | Y | 100 | M. haemophilum | Alive  Resolved |
| [58] | France | 57 | NA | FL | NA | NA | NA | Y | Skin | N | N | 330 | M. chelonae | Died  Other Cause |
| [58] | France | 57 | NA | Myelofibrosis | NA | NA | NA | Y | Skin | N | N | 540 | M. chelonae | Alive  Resolved |
| [59] | US | 7 | M | ALD | NA | NA | NA | NA | Blood | Y | N | 148 | M. immunogenum | NA |
| [59] | US | 10 | F | MDS | NA | NA | NA | NA | Pulmonary | N | Y | 502 | M. chelonae | NA |
| [59] | US | 11 | F | ALL | NA | NA | NA | NA | Pulmonary | N | Y | 108 | M. chelonae | NA |
| [59] | US | 15 | M | FA | NA | NA | NA | NA | GI | N | N | 1642 | M. chelonae | NA |
| [59] | US | 19 | F | HLH | NA | NA | NA | NA | Pulmonary | N | Y | 182 | M. chelonae | NA |
| [59] | US | 21 | M | HL | NA | NA | NA | NA | Pulmonary | N | Y | 730 | M. chelonae | NA |
| [60] | Japan | 11 | F | AA | 5/6 | NA | FLU/CY/TBI | Y | Cathether | N | N | 730 | MAC | Alive  Resolved |
| [61] | Australia | 55 | M | AML | NA | NA | NA | Y | Skin | N | N | 1460 | M. haemophilum | Died  Other Cause |
| [61] | Australia | 51 | M | AML | NA | NA | NA | Y | Skin | N | N | 1095 | M. chelonae | Alive  Resolved |
| [62] | Japan | 68 | F | AML | NA | NA | NA | Y | Pulmonary | N | Y | 2555 | MAC | Alive  Resolved |
| [63] | US | 57 | F | AML | 10/10 | NA | NA | Y | Skin | N | N | 480 | M. haemophilum | Alive  Resolved |
| [64] | US | 13 | M | IPEX | 10/10 | PBSC | FLU/MEL/ALEMT | Y | Lymph Nodes/GI/Liver | Y | N | 253 | M. genavense | Alive  Resolved |
| [65] | Pakistan | 15 | M | Thal | NA | NA | NA | NA | Lymph Nodes | N | N | 365 | M. abscessus | Alive  Resolved |
| [66] | Japan | 50 | M | CML | NA | NA | NA | NA | Pulmonary | N | Y | 3285 | M. abscessus | Alive  Resolved |
| [67] | Japan | 27 | M | ALL | NA | NA | NA | Y | Pulmonary | N | Y | 1530 | M. abscessus | Alive  Not resolved |
| [67] | Japan | 47 | M | ATL | NA | NA | NA | Y | Pulmonary | N | Y | 398 | M. abscessus | Alive  Resolved |
| [67] | Japan | 48 | M | ATL | NA | NA | NA | Y | Pulmonary | N | Y | 1890 | M. massiliense | Alive  Resolved |
| [68] | France | 66 | F | MDS | 10/10 | NA | BU/FLU/ATG | Y | Pulmonary | N | Y | 1095 | M. abscessus | Alive  Resolved |
| [69] | US | 6 | M | Farber disease | NA | UCB | NA | NA | Blood | Y | N | 180 | M. mucogenicum | Alive  Resolved |
| [69] | US | 14 | F | T cell chronic active EBV | 10/10 | NA | NA | NA | Catheter/blood | Y | N | 180 | M. immunogenum | Alive  Resolved |
| [70] | US | 30 | M | ALL | NA | NA | NA | Y | GI | N | N | 3650 | MAC | Alive  Resolved |
| [71] | US | 69 | M | AML | HAPLO | NA | NA | NA | Skin | N | N | NA | M. massiliense | Died  NTM Cause |
| [72] | Japan | 51 | M | MDS | NA | NA | NA | NA | Blood/Skin | Y | N | 3 | M. massiliense | Alive  Resolved |
| [73] | Australia | 59 | F | Myelofibrosis | 10/10 | NA | FLU/MEL/ATGE | N | Blood | Y | N | 29 | M. abscessus | Died  Other Causes |
| [74] | Japan | 62 | F | CMML | 10/10 | BM | TBI/BU/FLU | N | Blood,Muscles,Joint | Y | N | 570 | M. abscessus | Died  Other Causes |
| [74] | Japan | 46 | F | AML | 10/10 | PBSC | TBI/CY | N | Catheter | N | N | 12 | M. chelonae | Alive  Resolved |
| [74] | Japan | 21 | M | MPAL | haplo | PBSC | FLU/MEL/TBI/ARA-c | N | Blood | Y | N | 365 | M. chelonae | Died  Other Causes |
| [74] | Japan | 67 | M | AML | 10/10 | BM | BU/FLU/TBI | N | Blood | Y | N | 10 | M. abscessus | Alive  Resolved |
| [75] | Japan | 18 | M | ALL | 7/8 | BM | TBI/CY/VP16 | Y | Joint | N | N | 1270 | M. kansasii | Died  Other Causes |
| [24] | US | 13 | M | MDS | NA | NA | NA | NA | Skin | N | N | 73 | M. fortuitum | Alive  Resolved |
| [25] | US | 37 | M | ALL | NA | BM | NA | Y | Pulmonary | N | Y | 540 | MAC | Died  NTM Cause |
| [26] | US | 34 | F | CML | 10/10 | NA | NA | N | CVC/blood | Y | N | 54 | M. chelonae | Alive  Resolved |
| [26] | US | 12 | F | AA | 10/10 | NA | NA | N | Skin | N | N | 90 | M. fortuitum | Alive  Resolved |
| [26] | US | 4 | M | AML | 10/10 | NA | NA | N | Blood | Y | N | 50 | M. fortuitum | Alive  Resolved |
| [26] | US | 24 | F | CML | 10/10 | NA | NA | Y | CVC/blood | Y | N | 69 | M. fortuitum | Died  NTM Causes |
| [26] | US | 49 | M | CML | 10/10 | NA | NA | Y | CVC/blood | Y | N | 52 | M. fortuitum | Alive  Resolved |
| [26] | US | 29 | F | AML | 10/10 | NA | NA | N | CVC/blood | Y | N | 7 | M. chelonae | Alive  Resolved |
| [76] | US | 49 | F | AML | NA | NA | NA | NA | Pulmonary/Skin | Y | Y | 90 | M. haemophilum | Alive  Resolved |
| [76] | US | 32 | M | MDS | NA | NA | NA | NA | Pulmonary/Blood | Y | Y | NA | M. haemophilum | Died  NTM Causes |
| [76] | US | 27 | M | AA | NA | NA | NA | NA | Pulmonary | N | Y | NA | M. haemophilum | Died  NTM Causes |
| [76] | US | 30 | F | APL | NA | NA | NA | NA | Skin | N | N | NA | M. haemophilum | Alive  Resolved |
| [76] | US | 29 | F | AML | NA | NA | NA | NA | Synovial | N | N | NA | M. haemophilum | Alive  Resolved |
| [76] | US | 28 | F | APL | NA | NA | NA | NA | Skin | N | N | NA | M. haemophilum | Alive  Resolved |
| [76] | US | 43 | M | MDS | NA | NA | NA | NA | Pulmonary | N | Y | NA | M. haemophilum | Died  NTM Causes |
| [76] | US | 24 | M | AML | NA | NA | NA | NA | Skin | N | N | NA | M. haemophilum | Alive  Resolved |
| [76] | US | 33 | F | NHL | NA | NA | NA | NA | Skin | N | N | NA | M. haemophilum | Alive  Resolved |
| [76] | US | 42 | M | NHL | NA | NA | NA | NA | Skin | N | N | NA | M. haemophilum | Alive  Resolved |
| [76] | US | 49 | F | AML | NA | NA | NA | NA | Skin | N | N | NA | M. haemophilum | Alive_Resolved |
| [76] | US | 46 | M | CML | NA | NA | NA | NA | Skin | N | N | NA | M. haemophilum | Alive  Resolved |
| [76] | US | 42 | M | CML | NA | NA | NA | NA | Pulmonary | N | N | NA | M. haemophilum | Died  NTM Cause |
| [76] | US | 47 | F | AA | NA | NA | NA | NA | Synovial, Bone | N | N | NA | M. haemophilum | Alive  Resolved |
| [29] | China | 50 | M | Myeloma | 10/10 | NA | BU/CY | Y | Pulmonary | N | Y | 2040 | M. scrofulaceum | Alive  Resolved |
| [29] | China | 47 | F | CML | 10/10 | NA | BU/CY | N | CVC | N | N | 1620 | M. chelonae | Alive_Resolved |
| [29] | China | 43 | F | CML | 10/10 | NA | BU/CY | N | CVC/Blood | Y | N | 120 | M. gordonae | Alive  Resolved |
| [29] | China | 30 | F | AML | 10/10 | NA | BU/CY/TBI | Y | Pulmonary | N | Y | 450 | M. fortuitum | Died  NTM Cause |
| [29] | China | 27 | M | AML | 10/10 | NA | BU/CY | Y | Pulmonary | N | Y | 480 | M. chelonae | Alive  Resolved |
| [29] | China | 19 | M | ALL | 10/10 | NA | CY/TBI | Y | Pulmonary | N | Y | 330 | M. fortuitum | Died  NTM Cause |
| [29] | China | 21 | F | AML | 10/10 | NA | BU/CY | Y | Pulmonary | N | Y | 510 | M. chelonae | Died  NTM Cause |
| [29] | China | 56 | F | CML | 10/10 | NA | BU/CY | N | Skin/Sputum/urine | Y | Y | 1140 | M. fortuitum | Died  Other Cause |
| [20] | US | 45 | M | CML | 10/10 | NA | TBI/TT/ CY/ATG | N | Skin | N | N | 157 | M. haemophilum | Alive  Resolved |
| [20] | US | 35 | F | AML | 10/10 | NA | BU/CY | Y | Pulmonary | N | Y | 303 | M. xenopi | Died_  NTM Cause |
| [20] | US | 48 | F | MDS | 10/10 | NA | TBI/TT/CY/ATG | N | Skin | N | N | 259 | M. haemophilum | Alive  Resolved |
| [20] | US | 42 | M | AML | 10/10 | NA | TBI/TT/CY/ATG | N | Pulmonary | N | Y | 145 | M. haemophilum | Died  NTM Causes |
| [20] | US | 49 | F | AML | 10/10 | NA | TBI/TT/CY/ATG | N | Skin | N | N | 123 | M. haemophilum | Alive  Resolved |
| [20] | US | 24 | M | AML | 10/10 | NA | TBI/TT/CY/ATG | Y | Skin | N | N | 235 | M. haemophilum | Alive  Resolved |
| [20] | US | 33 | F | Anaplastic LCL | 10/10 | NA | TBI/TT/CY, ATG | N | Skin | N | N | 105 | M. haemophilum | Alive  Resolved |
| [20] | US | 36 | F | AML | 10/10 | NA | TBI/CY | N | Pulmonary | N | Y | 30 | MAC | Died  Other Causes |
| [20] | US | 32 | M | AML | NA | NA | TBI/CY/ Ara-C | Y | CVC/blood | Y | N | 167 | M. fortuitum and M. haemophilum | Died  NTM Causes |
| [20] | US | 30 | F | HL | 10/10 | NA | FLU/MEL | N | CVC/blood/Pulmonary | Y | Y | 85 | MAC | Alive  Resolved |
| [20] | US | 42 | M | low grade NHL | 10/10 | NA | TBI/TT/CY/ ATG | N | Skin | N | N | 108 | M. haemophilum | Alive  Resolved |
| [20] | US | 32 | M | MDS | 10/10 | NA | TBI/TT/ CY/ATG | Y | blood/stool/BM/liver | Y | N | 320 | MAC | Died  Other Causes |
| [20] | US | 29 | M | AA | 10/10 | NA | FLU/ATG | N | CVC/blood | Y | N | 178 | M. fortuitum | Alive  Resolved |
| [20] | US | 46 | F | AA | 10/10 | NA | TBI/ TT/CY/ ATG | N | Bone/Joint/BM | Y | N | 153 | MAC and M. haemophilum | Alive  Resolved |
| [20] | US | 32 | M | AML | 10/10 | NA | TBI/CY/ Ara-C | Y | Pulmonary | N | Y | 88 | M. abscessus | Alive  Resolved |
| [20] | US | 41 | F | low grade NHL | 10/10 | NA | TBI/TT/CY/ATG | Y | Pulmonary | N | Y | 1105 | MAC | Alive  Resolved |
| [31] | US | 4 | F | NBL/MDS | NA | UCB | NA | Y | GI | N | N | 14 | MAC | Died  NTM Causes |
| [31] | US | 1NA2 | NA | HLH | NA | UCB | NA | NA | Skin | N | N | 35 | M. kansasii | Alive  Resolved |
| [31] | US | 16 | M | CML | NA | NA | NA | NA | CVC/blood | Y | N | 130 | M. immunogenum | Alive  Resolved |
| [32] | US | 3 | F | NBL/MDS | NA | UCB | NA | Y | Blood | Y | N | 14 | MAC | Died  Other Causes |
| [32] | US | 19 | M | CML | 10/10 | NA | BLU/FLU/ALEMT | NA | CVC/blood | Y | N | 133 | M. immunogenum | Alive  Resolved |
| [32] | US | 13 | M | SAA | 10/10 | NA | CY/FLU/ ATG | Y | Pulmonary | N | Y | 269 | MAC | Alive  Resolved |
| [32] | US | 3 | F | CML | 10/10 | NA | BLU/FLU/ ALEMT | Y | Pulmonary | N | Y | 127 | MAC and M. gordonae | Alive  Resolved |
| [32] | US | 2 | M | HLH | NA | UCB | BU/CY/VP16/ALEMT | NA | Skin | N | N | 35 | M. kansasii | Alive  Resolved |
| [33] | Spain | 7 | F | ALL | 10/10 | NA | NA | Y | Pulmonary | N | Y | 120 | M. kansasii | Alive  Resolved |
| [33] | Spain | 9 | M | HLH | 10/10 | NA | NA | Y | Pulmonary | N | Y | 148 | M. kansasii | Alive  Resolved |
| [33] | Spain | 9 | M | ALL | 10/10 | NA | NA | Y | Pulmonary | N | Y | 365 | M. abscessus | DiedNTM Cause |
| [33] | Spain | 9 | M | MDS | 10/10 | NA | NA | N | Pulmonary | N | Y | 15 | MAC | Alive  Resolved |
| [21] | Canada | 39 | M | Myelofibrosis | NA | NA | NA | Y | Pulmonary | N | Y | 363 | MAC | Died_  Other Cause |
| [21] | Canada | 52 | F | ALL | NA | NA | NA | Y | Pulmonary | N | Y | 2798 | MAC | Died  Other Cause |
| [21] | Canada | 41 | F | Myelofibrosis | NA | NA | NA | Y | Pulmonary | N | Y | 2755 | MAC | Died  Other Cause |
| [21] | Canada | 56 | M | AML | NA | NA | NA | Y | Pulmonary | N | Y | 1147 | MAC | Died  Other Cause |
| [21] | Canada | 55 | F | Mantle cell lymphoma | NA | NA | NA | Y | Pulmonary | N | Y | 143 | MAC | Died  Other Cause |
| [21] | Canada | 50 | M | CLL | NA | NA | NA | Y | Pulmonary | N | Y | 224 | MAC | Died  Other Cause |
| [21] | Canada | 65 | F | MDS | NA | NA | NA | Y | Pulmonary | N | Y | 1873 | MAC | Died  Other Cause |
| [21] | Canada | 41 | F | AA | NA | NA | NA | Y | Pulmonary | N | Y | 170 | MAC | Alive  Resolved |
| [21] | Canada | 54 | F | AML | NA | NA | NA | Y | Pulmonary | N | Y | 543 | MAC | Died  Other Cause |
| [21] | Canada | 63 | M | CML | NA | NA | NA | Y | Pulmonary | N | Y | 343 | MAC | Died  Other Cause |
| [21] | Canada | 48 | F | AML | NA | NA | NA | Y | Pulmonary | N | Y | 742 | M. fortuitum | Died  Other Cause |
| [21] | Canada | 64 | M | AML | NA | NA | NA | Y | Pulmonary | N | Y | 664 | M. fortuitum | Died  Other Cause |
| [21] | Canada | 59 | M | AML | NA | NA | NA | Y | Pulmonary | N | Y | 272 | M. fortuitum | Alive  Resolved |
| [21] | Canada | 49 | M | AML | NA | NA | NA | Y | Pulmonary | N | Y | 429 | M. fortuitum | Alive  Resolved |
| [21] | Canada | 64 | M | NHL | NA | NA | NA | Y | Pulmonary | N | Y | 649 | M. xenopi | Died  Other Cause |
| [21] | Canada | 60 | M | AML | NA | NA | NA | NA | Pulmonary | N | Y | 310 | M. xenopi | Died  Other Cause |
| [21] | Canada | 45 | M | CLL | NA | NA | NA | Y | Pulmonary | N | Y | 133 | M. xenopi | Died  Other Cause |
| [21] | Canada | 36 | F | MDS | NA | NA | NA | Y | Pulmonary | N | Y | 356 | M. abscessus | Died  Other Cause |
| [21] | Canada | 45 | F | MDS | NA | NA | NA | Y | Pulmonary | N | Y | 730 | M. abscessus | Alive  Resolved |
| [21] | Canada | 51 | M | Myelofibrosis | NA | NA | NA | Y | Pulmonary | N | Y | 519 | M. abscessus | Died  Other Cause |
| [35] | Taiwan | 32 | M | ALL | 10/10 | NA | CY/TBI | Y | Pulmonary | N | Y | NA | Unclassified | Died  Other Cause |
| [35] | Taiwan | 35 | F | ALL | 10/10 | NA | CY/TBI | NA | Pulmonary | N | Y | NA | Unclassified | Alive  Resolved |
| [35] | Taiwan | 44 | M | ALL | 10/10 | NA | CY/TBI | Y | Pulmonary | N | Y | NA | Unclassified | Died  NTM Cause |
| [35] | Taiwan | 57 | F | ALL | 10/10 | NA | BU/FLU | NA | Joint /Spine | Y | N | NA | M. kansasii | Alive  Resolved |
| [35] | Taiwan | 25 | F | ALL | 10/10 | NA | CY/TBI | N | Pulmonary | N | Y | NA | Unclassified | Alive  Resolved |
| [35] | Taiwan | 23 | M | ALL | 10/10 | NA | CY/TBI | NA | Pulmonary | N | Y | NA | Unclassified | Alive  Resolved |
| [35] | Taiwan | 50 | M | AML | 10/10 | NA | BU/FLU | N | Pulmonary | N | Y | NA | Unclassified | Died  Other Cause |
| [35] | Taiwan | 24 | F | AML | 10/10 | NA | BU/CY | Y | Pulmonary | N | Y | NA | Unclassified | Died  Other Cause |
| [35] | Taiwan | 49 | F | AML | 10/10 | NA | BU/CY | NA | Pulmonary | N | Y | NA | Unclassified | Died  Other Cause |
| [35] | Taiwan | 55 | F | AML | 10/10 | NA | BU/CY | NA | Pulmonary | N | Y | NA | MAC | Alive  Resolved |
| [35] | Taiwan | 60 | M | AML | HAPLO | NA | BU/CY | NA | Pulmonary | N | Y | NA | Unclassified | Died  NTM Cause |
| [35] | Taiwan | 52 | F | AML | 10/10 | NA | BU/CY | NA | Pulmonary | N | Y | NA | M. chelonae | Alive  Resolved |
| [35] | Taiwan | 29 | F | AML | 10/10 | NA | BU/CY | NA | Pulmonary | N | Y | NA | Unclassified | Died  Other Cause |
| [35] | Taiwan | 28 | M | CML | 10/10 | NA | BU/FLU | NA | Pulmonary | N | Y | NA | Unclassified | Died  Other Cause |
| [35] | Taiwan | 50 | F | CML | 10/10 | NA | BU/FLU | N | Pulmonary | N | Y | NA | Unclassified | Died  Other Cause |
| [35] | Taiwan | 46 | M | NHL | 10/10 | NA | BU/FLU | NA | Pulmonary | N | Y | NA | Unclassified | Alive  Resolved |
| [35] | Taiwan | 53 | F | NHL | 10/10 | NA | BU/FLU | NA | Bone Marrow | N | N | NA | Unclassified | Died  NTM Cause |
| [35] | Taiwan | 26 | M | NHL | 10/10 | NA | CY/TBI | NA | Pulmonary | N | Y | NA | Unclassified | Alive  Resolved |
| [35] | Taiwan | 56 | M | MDS | 10/10 | NA | BU/CY | Y | Pulmonary | N | Y | NA | Unclassified | Alive  Resolved |
| [35] | Taiwan | 52 | M | MM | 10/10 | NA | BU/MEL | Y | Pulmonary | N | Y | NA | Unclassified | Alive  Resolved |
| [35] | Taiwan | 26 | F | NHL | 10/10 | NA | CY/TBI | NA | Pulmonary | N | Y | NA | Unclassified | Died  Other Cause |
| [27] | China | 4 | F | SAA | NA | NA | NA | Y | GI | N | N | 70 | M. chelonae | NA |

Abbreviations: AA, aplastic anemia; A/W, alive and well; AML, acute myeloid leukemia; ALL, acute lymphoblastic leukemia; ARA-c, cytarabine; ATG, anti-thymocyte globulin; BM, bone marrow; BU, Busulfan; CAMP, campath/alemtuzumab CY, Cyclophosphamide; CML, chronic myeloid leukemia; CVC, central venous catheter; F, female; Flu, Fludarabine; IPEX, immune dysregulation, polyendocrinopathy, enteropathy, X-linked; M, male; MDS, myelodisplastic syndrome; Mel, Melphalan; MM, multiple myeloma; N, no; NHL, non-hodgkin lymphoma; PBSC, peripheral blood stem cells; TBI, total body irradiation; Thio, Thiotepa; UCB, umbilical cord blood; US, United States; Y, yes, NA; not applicable.
